# Sex Differences of Sarcopenia in an Elderly Asian Population: The Prevalence and Risk Factors

**DOI:** 10.3390/ijerph191911980

**Published:** 2022-09-22

**Authors:** Jongseok Hwang, Soonjee Park

**Affiliations:** 1 Institute of Human Ecology, Yeungnam University, Gyeongsan 38541, Korea; 2 Department of Clothing and Fashion, Yeungnam University, Gyeongsan 38541, Korea

**Keywords:** sarcopenia, prevalence, risk factors, prevalence, odd ratio, the elderly

## Abstract

The loss of muscle mass is widespread in age-related health phenomena in the elderly population. This study examined the prevalence of sarcopenia in a community-dwelling elderly population according to gender. The study also identified gender-specific risk factors in older people aged 75–84 years old. One thousand two hundred and ninety-three participants aged between 75 and 84 years from the National Health and Nutrition Examination Surveys in Korea were investigated. The prevalence of sarcopenia in males and females in the weighted-value sample was 41.2% (95%CI: 35.8–46.8) and 37.2% (32.7–41.9), respectively. Gender-specific clinical risk factors in males were height, weight, body mass index, waist circumference, skeletal muscle mass index, fasting glucose, and triglyceride levels. Height, weight, body mass index, waist circumference, skeletal muscle mass index, and total cholesterols were clinical risk factors for females. These outcomes would be crucial to primary care clinicians and health care professionals when patients require a referral for early detection and treatment. Health care professionals and clinicians can quickly identify potential sarcopenic patients by acknowledging the gender-specific prevalence and risk factors.

## 1. Introduction

The loss of muscle mass is a widespread age-related health phenomenon in the elderly [1]. The loss of muscle is a progressive and generalized skeletal muscle disorder that brings about adverse outcomes, including falls, fractures, physical disability, dyslipidemia, metabolic disorder, quality of life impairments, and mortality. Since Critchley first described muscle atrophy and weakness in 1931 [2], sarcopenia has become a significant challenge for the health of older people.

The elderly population of more than 65 years old has grown dramatically in Asia. Korea is one of the fastest aging countries in Asia. The older population is expected to reach 19,000,000 (40% of the whole population) in 2050 from 5,370,000 (15.7%) in 2022 [3]. Diseases associated with aging, such as sarcopenia, have more influence in Korea and Asia.

On the other hand, numerous sarcopenic research studies have classified subjects into a single group, even though the body composition and condition of the older population differ according to age. Therefore, classifying the elderly population according to age is essential for investigating the characteristics of sarcopenia properly. The elderly population should be divided into young old (65–74), old (75–84), and oldest-old (85+) groups [3,4,5].

In particular, aging progresses rapidly in the age range of 75–84 years because of changes in the following: cardiorespiratory function [6], the neuromuscular system [7], hormone function [8], cytokine milieu, mitochondrial dysfunction and oxidative stress, myogenic capacity, and protein turnover [9]. Specifically, the cardiorespiratory function regarding an individual’s peak oxygen consumption and forced expiratory volume critically decreased [6]. In the neuromuscular system, subjects with a mean age of 82 years showed a 61% reduction in the number of motor units of the tibialis anterior muscle compared to young adults [7]. Furthermore, the elderly, with a mean age of 79.2 years, showed decreased total testosterone levels with low muscle strength and physical performance [8]. These series of changes with aging deteriorate the physical acidity in the activity of daily life. In turn, the reduced activity increases the risk of sarcopenia, osteoporosis, and subjective lower well-being, as well as the life expectancy by 0.68 years [10]. In this way, sarcopenia accelerates between 75 and 84 years due to aging. Prevalence and risk factor studies are essential, but studies in this age group are insufficient.

The sarcopenia risk factors are rarely recognized or diagnosed by primary care health professionals, even though sarcopenia is recognized as an internationally classified disease with an ICD-10-CM code. The average general practitioner has between 7 and 10 min for each patient visit. For a primary care clinician to consider making a referral for the diagnosis and treatment for sarcopenia, they need to recognize that there is a likelihood that the individual may be sarcopenic [11]. The lack of knowledge of the average clinician about the existence of sarcopenia as a disease further increases the likelihood that the diagnosis will be missed [12]. Sarcopenia is commonly marked and presents no specific symptoms until it is serious. Knowing the fundamental characteristics of the risk factors related to early detection and prevention is crucial [13]. The early diagnosis of sarcopenia focuses on detecting symptomatic patients as early as possible. Once a sarcopenia diagnosis and intervention are delayed or missed, greater problems related to treatment, poor quality of life, and higher care costs could occur.

There is a discrepancy in gender-driven risk factors for sarcopenia among related studies [14,15,16,17,18]. Furthermore, several epidemiological studies by sex mentioned an inconsistency regarding prevalence [15,19,20,21,22,23]. They reported that gender-specific differences in the absolute muscle loss rates for men are larger than for women, which may not be accredited to the greater initial muscle mass in men [21,24,25].

Although there is a lower incidence of sarcopenia in women, a previous study showed that sarcopenia is associated with greater mortality in women. This casts doubt on the possible differential sex-specificity that brings about sarcopenia, necessitating a comprehensive understanding of the inherent mechanism [26]. Health professionals and clinicians should know the prevalence and risk factors according to gender, which helps develop novel therapeutic agents for predicting, preventing, and treating sarcopenia.

Therefore, this study examined the prevalence of sarcopenia in the community-dwelling elderly population according to gender and identified the gender-specific risk factors in the elderly aged between 75 and 84 years old. The present study proposes two hypotheses. A specific prevalence of sarcopenia exists in older populations according to gender. There would be gender-specific risk factors in the community-dwelling elderly population with sarcopenia.

## 2. Materials and Methods

### 2.1. Study Population and Design

The present study was based on data collected from the Korea National Health and Nutrition Examination Survey (KNHANES). The KNHANES is a survey with a stratified, clustered, multistage probability sampling design to monitor the health-risk behaviors of the population and is conducted by the Center for Disease Control and Prevention. Three thousand seven hundred and thirty-seven people participated in this survey during 2008–2011. Of these, the data of 1287 participants were included in the final analysis for older adults aged between 75 and 84 years old. Among the 1287 participants, 484 elderly participants were assigned to a sarcopenia group, and the remaining 809 participants were assigned to a normal group. The criterion for dividing participants between the sarcopenia and normal groups depended on a skeletal muscle mass index score. The present study was approved by the institutional review board of the Center for Disease Control and Prevention (IRB number: 2008-04EXP-01-C, 2009-01CON-03-2C, 2010-02CON-21-C and 2011-02CON-06-C). An informed written consent form was obtained from the research participants [Table 1].

### 2.2. Study Variables

#### 2.2.1. Anthropometric Measure and Skeletal Muscle

To take the anthropometric measurements, all subjects removed their shoes, socks, hat, and hairpins and wore light clothes. The participants’ height and weight were gauged using calibrated automatic body measurement equipment and were recorded to the nearest 0.1 cm or kg. The body mass index (BMI) was calculated as weight (kg) divided by height squared (m^2^). The waist circumference (WC) was assessed to the nearest 0.1 cm in a horizontal plane at the midline between the last rib and the iliac crest at the end of a normal expiration. The skeletal muscle mass index (SMI) was calculated as ASM (kg)/BMI (kg/m^2^). The appendicular skeletal muscle mass (ASM) was assessed by dual X-ray absorptiometry (QDR4500A; Hologic, Inc., Bedford, MA, USA).

#### 2.2.2. Blood Tests, Blood Pressure, and Other Survey Variables

The fasting glucose (FG), triglyceride, and total cholesterol (TC) values were obtained from a blood test. Blood was drawn from the non-dominant arm that is not used frequently for blood tests after fasting for at least eight hours. During blood collection, the coagulation promoter and blood were mixed well, and the collected blood was centrifuged. The pre-processing of blood collection was carried out immediately after blood collection in the mobile examination vehicle. All tests were analyzed within 24 h of the sample collection.

The systolic blood pressure (SBP) and diastolic blood pressure (DBP) were measured by a well-trained practitioner using a mercury sphygmomanometer. A blood pressure cuff was located at the heart level, while the subjects sat in a chair after at least five minutes of rest.

Variables collected by the survey were age, smoking status, and drinking status. Cigarette smokers and alcohol drinkers were designated into three domains: non-user, ex-user, or current user.

### 2.3. Determination of Sarcopenia

Sarcopenia, for which the disease code is ICD-10-CM (M62.84), is diagnosed by appendicular skeletal muscle mass. The appendicular skeletal muscle mass (ASM) was assessed through dual X-ray absorptiometry (QDR4500A; Hologic, Inc., Bedford, MA, USA). The skeletal muscle mass index (SMI) was calculated as ASM (kg)/ BMI (kg/m^2^), and sarcopenia was defined as an SMI of <0.789 in men and <0.521 in women based on the criteria of the Foundation for the National Institutes of Health Sarcopenia Project [27].

### 2.4. Statistical Analysis

All analyses were performed using Statistical Package for the Social Sciences Windows version 22.0 (SPSS Inc, Chicago, IL, USA). The descriptive data are presented as the mean ± standard deviation. Complex sampling analysis was conducted by adjusting the weights provided by the National Health and Nutrition Examination Survey in Korea. Independent *t*-tests and chi-square analyses were performed to compare the variables of the two groups. Multiple logistic regression with adjusted covariates was used to predict sarcopenia, calculating the odds ratio of the sarcopenic risk factor in both genders. The statistical significance was set to 0.05 for all variables.

## 3. Results

### 3.1. Prevalence

The weighted value of sarcopenic prevalence in men and women was 41.2% (95%CI: 35.8–46.8) and 37.2% (32.7–41.9), respectively. The prevalence in sarcopenic males was greater than in females. Table 2 lists the gender-specific prevalence of sarcopenia.

### 3.2. Gender-Specific Risk Factors

#### 3.2.1. Anthropometric Measure and Skeletal Muscle Index Variables

The risk factors in both males and females in terms of height, weight, BMI, WC, and SMI were statistically significant (*p* < 0.05). Table 3 lists the gender-specific risk factors in the anthropometric measures and skeletal muscle index variables.

#### 3.2.2. Blood Tests, Blood Pressure, and Other Survey Variables

The risk factors in men, including the FG and triglyceride levels, were statistically significant (*p* < 0.05), while TC, SBP, DBP, age, SS, and DS were not significant (*p* > 0.05). The significant risk factor for women was TC. Other variables, such as FG, triglyceride, SBP, DBP, age, SS, and DS, were not statistically significant (*p* > 0.05). Table 4 lists the gender-specific risk factors in blood tests, blood pressure, and other survey variables.

#### 3.2.3. Odd Ratio for Sarcopenia in Males

Each multiple logistic regression analysis was performed by gender to identify the odds ratio. The height, weight, BMI, WC, SMI, and triglyceride variables showed statistical significance (*p* < 0.01), and the respective values were 0.003 (0.000–0.023), 2.290 (0.118–44.417), 4.821(1.797–9.453), 0.366 (0.246–0.543), 0.102 (0.017–0.519), and 1.102 (0.897–1.356). FC is not statistically significant (*p* > 0.05) [Table 5].

#### 3.2.4. Odd Ratio for Sarcopenia in Females

The height, weight, BMI, WC, SMI, and TC variables showed statistical significance (*p* < 0.01) with values of 0.001 (0.000–0.004), 2.291 (0.903–2.803), 1.177 (0.751–18.44), 1.230 (0.984–1.537), 0.387 (0.208–0.719), and 1.233 (1.171–1.297), respectively [Table 6].

## 4. Discussion

The present study evaluated the gender-specific prevalence and risk factors in the community-dwelling elderly population aged between 75 and 84. The sarcopenic prevalence was 41.2 (35.8–46.8)% in men, which was higher than the 37.2 (32.7–41.9)% prevalence in women. This finding is consistent with studies conducted in the United States, China, and Hong Kong [28,29,30,31]. Brown et al. evaluated 4425 members of the older population in the United States and reported a male and female prevalence of 44.8% and 30.24%, respectively [28]. Similarly, Liu et al. investigated 4500 Chinese urban community-dwelling older people. They reported that the prevalence of sarcopenia in men and women was 22.1% and 17.8%, respectively [31]. Hai et al. assessed 834 community-dwelling Chinese individuals and reported that the incidence of sarcopenia was 11.3% in men and 9.8% in women [30]. Chan et al. reported 9.30% in males and 5.30% in females out of 3957 elderly people in Hong Kong [29]. Overall, these studies reported a higher prevalence of sarcopenia in elderly males than females.

A possible underlying reason for the higher prevalence in men is related to the testosterone and insulin-like growth factor-1 levels of men. At the early stage of aging, women lose their muscle mass and strength more quickly than men due to menopause and the diminished estrogen level in the blood [32]. Nevertheless, in a further stage of aging, a deficit of insulin-like growth factor-1 and testosterone hormone levels appears in men’s blood, causing a higher speed of muscle function and mass loss, resulting in sarcopenia [33].

This finding contrasts with several studies [34,35]. Dam et al. investigated US community-dwelling elderly people and reported a sarcopenia incidence of 5.10% in males and 11.80% in females [34]. Hunt et al. reported 10.34% in men and 16.56% in women after investigating 1921 people in a Japanese community-dwelling elderly population [35]. The possible theoretical rationale for a higher prevalence of sarcopenia in elderly females than in elderly men has been attributed to higher dietary protein intake in Japan and the US [36,37]. A lack of dietary protein intake is considered to be a key factor contributing to the progressive loss of muscle mass [38]. The higher consumption of dietary protein in US and Japanese males might have promoted muscle protein synthesis, preventing sarcopenia. 

Regarding risk factors in anthropometric measures, waist circumference is a risk factor for sarcopenia in men and women. The outcome is consistent with several sarcopenic risk factor studies [28,39,40]. A population-based cohort study from the US National Health and Nutrition Survey identified that the odds ratios were 1.39 (1.05–1.84) in men and 1.44 (1.04–2.00) in women (95% of confidence interval) [28]. A Brazilian sarcopenic elderly population cohort study revealed an odds ratio of 17.90 (1.48–201.16) (95% CI) for the anthropometric indicators, such as waist circumference in males and females [39]. A Japanese community-dwelling elderly study suggested that the elderly population with sarcopenia has a larger waist circumference than the non-sarcopenic elderly population [40]. The possible theoretical rationale for the higher waist circumference in males and females is that the relationship between enhanced fat mass and lower muscle mass is mutually interdependent [41]. Individuals with sarcopenia frequently have problems with muscle power and function caused by muscle loss. The decreased muscle power and function cause poor physical exercise and performance, such as sitting to stand and walking long distances in indoor and outdoor settings [42]. Such a decreased physical activity level is strongly associated with decreased total daily energy expenditure and fat stores in the visceral and belly areas, enlarging the waist volume [42]. In contrast, a high-fat volume, especially visceral fat, produces large amounts of pro-inflammatory cytokines [43], interleukin 6, and C-reactive protein, which impedes the anabolic response of muscle tissue. Therefore, the decreased muscle and enlarged fat mass are interdependent [44].

High fasting glucose is another risk factor for sarcopenia in males. This result aligns with several sarcopenic studies [14,45,46,47]. Bersemi et al. conducted a sarcopenic cohort study on 157 sarcopenic community-dwelling elderly participants. They reported that the elderly population with sarcopenia showed a higher fasting glucose level than the counterpart group [46]. In addition, a Turkish sarcopenia study with 147 participants showed that sarcopenic patients have difficulty controlling their blood glucose levels [45]. A possible theoretical mechanism for higher fasting glucose levels in the sarcopenic group is that muscle mass is essential to regulate the blood glucose level after meals. The skeletal muscle stores approximately 80% of postprandial glucose after ingestion, preventing hyperglycemia from the blood. At the age of 80, only 60% of the skeletal muscle mass remains compared to people in their twenties [48]. Males are especially less sensitive to insulin than females because they have decreased glucose uptake by skeletal muscle [49]. This phenomenon occurs due to a lower proportion of type I muscle fiber and capillary density which are prone to insulin action in males [50]. Thus, decreased muscle is less likely to lead to an uptake in blood glucose, which accelerates hyperglycemia in the blood in elderly men.

Another risk factor for men is triglyceride levels. This factor is consistent with previous sarcopenic studies [51,52,53]. Du et al. [52] conducted a cross-section study on community-dwelling elderly individuals in East China, pointing out that the male sarcopenia group had increased serum triglycerides levels. Lu et al. [53] assessed 600 northern Taiwan community-dwelling elderly individuals and revealed that the sarcopenia group had significantly higher levels of triglyceride. Similarly, Buchmann et al. [51] investigated 1420 of the elderly population in Berlin and concluded that triglyceride levels were greater in the sarcopenic group than in the non-sarcopenic group. Total cholesterol is a risk factor for sarcopenia in women. The outcome was parallel with earlier sarcopenia studies [40,52]. Du et al. [52] report total cholesterol levels in the female sarcopenic group are higher than the female counterpart normal group. Sanada and her colleagues evaluated a Japanese population group of 1488 and mentioned that the total cholesterol values in those with sarcopenia are significantly higher than the normal group [40]. A possible underlying mechanism for such high levels of triglycerides and total cholesterol is related to insulin resistance [54] and high volumes of inflammatory cytokines [55].

Interestingly, the triglyceride levels in the sarcopenic male group are approximately equal to the same levels of the normal female group. A plausible underlying reason is that the larger amount of skeletal muscle in men plays a key role in promoting triglyceride-rich lipoprotein hydrolysis [56,57]. The skeletal muscle is an important organ to regulate systemic energy metabolism. Skeletal muscle mass leads to improvements in energy use by promoting TG-rich lipoprotein hydrolysis through increases in circulating lipoprotein lipase and glycosylphosphatidylinositol-anchored high-density lipoprotein binding protein 1 concentrations [57]. These processes cause lower levels of triglycerides in men compared to women. Therefore, the triglyceride levels of both the sarcopenic male group and the normal female group are almost the same.

Dual-energy X-ray absorptiometry is expensive and sometimes hard to access in primary clinical practice, though it is the gold standard, and its validity and reliability are well-recognized [58]. For this reason, primary clinicians and health care professionals are able to detect sarcopenia via the clinical risk factors in this research. Furthermore, we recommend using grip strength [59,60] and the strength, assistance with walking, rising from a chair, climbing stairs, and falls (SARC-F) questionnaire, which is a quick screening tool that can be implemented by health care professionals and clinicians in a short time [61,62] in order to diagnose potential sarcopenic patients.

The strong point of the present study is that it provides the first clinical evidence related to the prevalence and clinical risk factors according to gender in the elderly sarcopenic population. Nevertheless, the study has several limitations that need to be considered for future studies. First, the study did not consider sarcopenic obesity. The outcomes in fasting glucose and total cholesterol could be clearly understood in the elderly with sarcopenia by considering sarcopenic obesity. The second limitation is that nutrition and nutritional-related biomarkers of sarcopenia, including albumin, leptin, pre-albumin, 3-methylhistidine, creatinine, and cystatin C, which are sarcopenic risk factors, were not addressed in this study, though nutritional status was a key element for the diagnosis of sarcopenia. Creatinine and cystatin C are especially well known for being simple, economical and effective screening risk factors for sarcopenia. The third limitation is that the study did not present correlations between sex hormones and sarcopenia. Had estrogen and testosterone levels been explained, the gender differences would have been more clearly indicated. Finally, the study design was a cross-section design. The study could be more robust if it were a longitudinal design that evaluated the same sarcopenic population repeatedly to assess gender-specific risk factors.

## 5. Conclusions

The present study provides the first clinical evidence of the prevalence of sarcopenia and clinical risk factors according to gender in the sarcopenic elderly population. The rate of incidence of sarcopenia in men and women was 41.2% (95%CI: 35.8–46.8) and 37.2% (32.7–41.9), respectively, showing that males have a greater prevalence of sarcopenia than females. The clinical risk factors in males were height, weight, body mass index, waist circumference, skeletal muscle mass index, fasting glucose, and triglyceride levels. Height, weight, body mass index, waist circumference, skeletal muscle mass index, and total cholesterols were the clinical risk factors for females. The discovery of gender-specific risk factors in the elderly would be very beneficial for making an early diagnosis and intervention to treat sarcopenia. In particular, these outcomes would be crucial to primary care clinicians and health care professionals when a patient requires a referral for early detection and treatment. Health care professionals and clinicians can quickly identify potential sarcopenic patients by acknowledging the gender-specific prevalence and risk factors.

## Figures and Tables

**Table 1:** General characteristics of the participants (n = 1293).

Variables	Sarcopenic Group (n = 484)	Normal Group (n = 809)	*p*
Sex (men/women) (%)	41.12/58.88	38.20/61.80	0.289
Age	77.98 ± 1.927	77.82 ± 1.918	0.162 **
Height (cm)	151.37 ± 8.945	155.74 ± 9.089	0.000 **
Weight (kg)	56.38 ± 9.692	53.57 ± 9.627	0.000 **
Body mass index (kg/m^2^)	24.53 ± 3.208	22.00 ± 2.939	0.000 **
Waist circumference (cm)	56.38 ± 9.692	53.57 ± 9.627	0.000 **
Skeletal muscle index (kg/m^2^)	0.575 ± 0.130	0.703 ± 0.149	0.000 **
Smoking status (%) (current-/ex-/non-smoker)	37.6/28.9/33.5	39.3/26.1/34.6	0.846
Drinking status (%) (current-/ex-/non-drinker)	28.9/14.6/56.5	30.2/13.1/56.8	0.337
Fasting glucose (mg/dL)	108.09 ± 27.959	101.44 ± 26.116	0.000 **
Triglyceride (mg/dL)	150.83 ± 78.390	132.17 ± 84.504	0.000 **
Total cholesterol (mg/dL)	197.96 ± 40.476	189.03 ± 35.770	0.000 **
Systolic blood pressure (mmHg)	134.62 ± 18.580	133.20 ± 18.017	0.175
Diastolic blood pressure (mmHg)	75.44 ± 10.282	74.56 ± 10.247	0.136

Values are the means ± SD. *p*-values according to an independent t-test and chi-square were significant between the two groups (** *p* < 0.01).

**Table 2:** Prevalence of sarcopenia according to gender.

	Men			Women		
	Sarcopenic Group (n = 119)	Normal Group (n = 309)	Total	Sarcopenic Group (n = 285)	Normal Group (n = 500)	Total
Un-weighted (%)	27.8	72.2	100	36.3	63.7	100
Weighted (%)	41.2 (35.8–46.8)	58.8 (53.2–64.2)	100	37.2 (32.7–41.9)	62.8 (58.1–67.3)	100

Weighted values are presented as the 95% confidence interval.

**Table 3:** Gender-specific risk factors in the anthropometric measures and skeletal muscle index variables.

	Men			Women		
	Sarcopenic Group (n = 119)	Normal Group (n = 309)	*p*	Sarcopenic Group (n = 285)	Normal Group (n = 500)	*p*
Height (cm)	159.981 ± 5.425	165.128 ± 5.028	0.000 **	145.362 ± 5.231	149.943 ± 5.468	0.000 **
Weight (kg)	60.692 ± 9.426	58.853 ± 9.086	0.029 *	53.369 ± 8.700	50.301 ± 8.434	0.000 **
BMI (kg/m^2^)	23.623 ± 2.819	21.531 ± 2.816	0.005 **	25.171 ± 3.313	22.289 ± 2.979	0.000 **
WC (cm)	86.557 ± 8.767	80.545 ± 9.778	0.000 **	85.769 ± 9.272	78.941 ± 9.289	0.000 **
SMI (kg/m^2^)	0.723 ± 0.050	0.877 ± 0.069	0.000 **	0.471 ± 0.037	0.596 ± 0.057	0.000 **

BMI: body mass index, WC: waist circumference, SMI: skeletal muscle index, values are means ± SD. *p*-value according to independent t-test and chi-square were significant between the two groups (* *p* < 0.05, ** *p* < 0.01).

**Table 4:** Gender-specific risk factors in blood tests, blood pressure, and other survey variables.

	Men			Women		
	Sarcopenic Group (n = 119)	Normal Group (n = 309)	*p*	Sarcopenic Group (n = 285)	Normal Group (n = 500)	*p*
FG (mg/dL)	109.587 ± 25.756	98.745 ± 22.121	0.000 **	107.000 ± 9.471	103.244 ± 28.356	0.123
Triglyceride (mg/dL)	146.368 ± 83.191	114.390 ± 63.986	0.000 **	154.047 ± 74.771	144.049 ± 94.015	0.178
TC (mg/dL)	183.613 ± 36.454	178.055 ± 32.703	0.106	208.312 ± 40.148	196.371 ± 35.890	0.000 **
SBP (mmHg)	134.005 ± 18.852	130.955 ± 18.411	0.072	135.056 ± 18.408	134.586 ± 17.647	0.742
DBP (mmHg)	74.995 ± 10.734	74.331 ± 10.282	0.468	75.750 ± 9.961	74.700 ± 10.233	0.164
Age (years)	77.864 ± 1.994	77.618 ± 1.933	0.816	78.053 ± 1.879	77.946 ± 1.899	0.448
SS (%) (current-/ex-/non-smoker)	53.6/28.6/17.8	55.4/29.2/15.4	0.098	11.6/4.7/83.6	14.3/2.9/82.9	0.202
DS (%) (current-/ex-/non-drinker)	52.1/37.1/10.8	56.9/28.8/14.3	0.521	27.5/23.2/49.3	28.2/24.5/47.3	0.760

SS: smoking status, DS: drinking status, FG: fasting glucose, TC: total cholesterol, SBP: systolic blood pressure, DBP: diastolic blood pressure; values are means ± SD or %. *p*-value according to independent t-test and chi-square were significant between the two groups (** *p* < 0.01).

**Table 5:** Odds ratio for sarcopenia in males.

Variables	OR (95% of CI)	*p*
Height	0.003 (0.000–0.023)	0.000 **
Weight	2.290 (0.118–44.417)	0.000 **
BMI	4.821 (1.797–9.453)	0.000 **
WC	0.366 (0.246–0.543)	0.000 **
SMI	0.102 (0.017–0.519)	0.000 **
FC	1.020 (0.899–1.157)	0.757
Triglyceride	1.102 (0.897–1.356)	0.000 **

Odds ratio (OR) values are presented as the 95% confidence interval (CI); multiple logistic regression performed (** *p* < 0.01).

**Table 6:** Odds ratio for sarcopenia in females.

Variables	OR (95% CI)	*p*
Height	0.001 (0.000–0.004)	0.000 **
Weight	2.291(0.903–2.803)	0.000 **
BMI	1.177(0.751–18.44)	0.000 **
WC	1.230(0.984–1.537)	0.000 **
SMI	0.387 (0.208–0.719)	0.000 **
TC	1.233 (1.171–1.297)	0.000 **

Odds ratio (OR) values are presented as the 95% confidence interval (CI); multiple logistic regression performed (** *p* < 0.01).

## Data Availability

All data were anonymized and can be downloaded from the website at https://knhanes.kdca.go.kr/knhanes (accessed on 1 August 2022).
